# Philadelphia Medical Society

**Published:** 1844-12-14

**Authors:** 


### RECORD OF MEDICAL SCIENCE.

### PHILADELPHIA MEDICAL SOCIETY.

This time-honored association for the promotion of medical science has commenced its winter sessions in good earnest. Interesting discussions have occurred, we learn, at every meeting, and on several evenings valuable papers have been read, among which was the Annual Oration, by Dr. Condie, and a paper entitled "*Observations on the Inapplicability of the term Congestion to the Malarial fevers of the South and West,*" by Dr. Isaac Parrish.

The Society having resolved to publish Dr. Condie's Oration, we shall defer any observations upon it until we are furnished with a copy.

We subjoin a brief abstract of some of the prominent points discussed in the paper of Dr. Parrish, and of the general conclusions stated.

First. That the disease termed congestive fever, is that form of intermittent and remittent fevers of malarial districts, formerly known under the appellation of malignant; differing only in degree from these fevers, and occurring in all regions where autumnal fevers prevail, being epidemic in the southern and south western sections of the United States, and generally appearing with most intensity along the low grounds skirting the rivers.

Secondly. That the symptoms which characterize this variety of malarial fever, and which have been attributed by some writers to congestion, viz.: the peculiar irregular, sighing respiration, sense of suffocation, præcordial uneasiness, intense thirst, obstinate vomiting, bloody or serous discharges—and where the brain is involved, stupor or delirium, &c. &c., do not necessarily indicate any peculiar engorgement of the several organs whose functions are disturbed, but that they are more properly attributable to a sudden and alarming diminution of nervous power.

Thirdly. That many of these phenomena supposed to indicate congestion of vital organs, bear a strong analogy to the effects produced by Le Gallois, Dupuytren and others, by the tying of the pneumogastric nerves in animals, and are more rationally explained upon the principle of diminished nervous power. And that, moreover, they occur in other conditions of the system, the reverse of congestion—as from copious uterine hemorrhages, after severe and mortal accidents attended with great loss of blood, in anemia, &c. &c.

Fourthly. That some of the symptoms referred to congestion may depend on the altered condition of the blood itself, in connection with the atony of the tissues, through which it passes.

Fifthly. That admitting the existence of passive congestion of the heart, vena cava, lungs, portal circle, &c., from a want of nervous power in the central organs to propel the blood to the remote vessels, still the term *congestive*, as applied to this disease, is inappropriate—because it designates a result or consequence of a primary cause, without indicating the cause itself—a result not peculiar to the disease in question, as it occurs to a greater or less extent in all cases of sudden nervous shock; and is, in fact, one of the phenomena of the dying state.

Sixthly. The therapeutical effect of remedies furnishes a strong argument in favor of the doctrine of *nervous prostration*, and not of *congestion*. General bleeding, which it was contended would be the great reliance of the physician in a disease of active congestion of vital organs, is admitted by the most experienced practitioners in this disease to have been prejudicial, while the use of powerful revulsives, stimuli, and particularly of quinia, (one of the most concentrated and active of the nervous stimulants,) are generally acknowledged to be the main remedies in controlling the violence of the symptoms, and in rescuing the patients.

Seventhly. That the use of names or terms have a powerful influence in forming the opinions, and regulating the practice of medical men—and hence that medical nomenclature should be based, as far as possible, on pathological principles—and should be descriptive of the true condition of the organ or organs peculiarly affected in a particular complaint. The importance of this consideration was particularly urged, where the adoption of the depletory or stimulant practice may depend upon it.

Eighthly. Applying the above principles to the disease in question, it was contended that, considering depression of the nervous power as the first link in the chain of morbid associations, in this, as in all other varieties of fever, a term should be used as a prefix to the words intermittent or remittent fever, which should represent the extraordinary degree of prostration peculiar to this form of fever—a term applicable to the nervous system, and not to the circulating system. Dr. Parrish suggested the word adynamic, as more expressive of the true pathology of this disease, than any other which had occurred to him.

The paper will probably appear in full at some future period. The above is a mere abstract.

